# Utilization of combined remote sensing techniques to detect environmental variables influencing malaria vector densities in rural West Africa

**DOI:** 10.1186/1476-072X-11-8

**Published:** 2012-03-23

**Authors:** Peter Dambach, Vanessa Machault, Jean-Pierre Lacaux, Cécile Vignolles, Ali Sié, Rainer Sauerborn

**Affiliations:** 1 Institute of Public Health, University of Heidelberg, Heidelberg, Germany; 2 Observatoire Midi Pyrénées/Laboratoire d'Aérologie, Toulouse, France; 3 Centre National d'Etudes Spatiales (CNES), Toulouse, France; 4 Centre de Recherche en Santé de Nouna, Nouna, Burkina Faso; 5 Centre for Global Health Research, Umeå University, Umeå, Sweden

**Keywords:** Remote sensing, High spatial resolution, SPOT 5 satellite, Malaria, Rural West Africa, Burkina Faso, Geographic information system, Digital elevation model, MODIS, TRMM

## Abstract

**Introduction:**

The use of remote sensing has found its way into the field of epidemiology within the last decades. With the increased sensor resolution of recent and future satellites new possibilities emerge for high resolution risk modeling and risk mapping.

**Methods:**

A SPOT 5 satellite image, taken during the rainy season 2009 was used for calculating indices by combining the image's spectral bands. Besides the widely used Normalized Difference Vegetation Index (NDVI) other indices were tested for significant correlation against field observations. Multiple steps, including the detection of surface water, its breeding appropriateness for *Anopheles *and modeling of vector imagines abundance, were performed. Data collection on larvae, adult vectors and geographic parameters in the field, was amended by using remote sensing techniques to gather data on altitude (Digital Elevation Model = DEM), precipitation (Tropical Rainfall Measurement Mission = TRMM), land surface temperatures (LST).

**Results:**

The DEM derived altitude as well as indices calculations combining the satellite's spectral bands (NDTI = Normalized Difference Turbidity Index, NDWI Mac Feeters = Normalized Difference Water Index) turned out to be reliable indicators for surface water in the local geographic setting. While *Anopheles *larvae abundance in habitats is driven by multiple, interconnected factors - amongst which the NDVI - and precipitation events, the presence of vector imagines was found to be correlated negatively to remotely sensed LST and positively to the cumulated amount of rainfall in the preceding 15 days and to the Normalized Difference Pond Index (NDPI) within the 500 m buffer zone around capture points.

**Conclusions:**

Remotely sensed geographical and meteorological factors, including precipitations, temperature, as well as vegetation, humidity and land cover indicators could be used as explanatory variables for surface water presence, larval development and imagines densities. This modeling approach based on remotely sensed information is potentially useful for counter measures that are putting on at the environmental side, namely vector larvae control via larviciding and water body reforming.

## Background

Malaria is still widespread in Western Africa and results in severe illness, death and hence in economic damage to households and national economy. The desirable overall use of countermeasures and strategies against malaria and its vector, such as use of bed nets, larviciding, habitat reduction etc., is still below the needed amount for showing a remarkable impact.

During the past two decades, remotely sensed data has been used to describe and predict geographical and temporal patterns in vector-borne disease transmission and disease prevalence [1-3]. The basic idea behind the remotely sensed assessment of malaria determinants is to define environmental parameters that can be used to identify areas with increased risk. One of the main goals of this approach could be the detection of breeding habitats or the mapping of vector densities through remote sensing techniques, while some other studies linked climate and environmental parameters directly to malaria prevalence [2,4-6]. The suitability of habitats for mosquito larvae breeding is dependent on the presence and distribution of specific environmental variables (*i.e*., surface water, water related vegetation and distribution and amount of precipitation) [7]. Studies mapping *Anopheles *mosquito breeding habitats, transmission, or disease have been made in Africa [4,8-10] South and Central America [11-14] and Asia [15,16]. Reliable information about vector density and malaria transmission risk is essential for understanding variations in disease epidemiology and targeting intervention programs, which are useful tools at the continental and national scales, but are less appropriate in a context of local-scale variations in disease patterns that often vary within a few kilometers distance. Nevertheless, high local variation in malaria epidemiology is particularly common in the Sahel region of Africa, where malaria is characterized by very focal and seasonal transmission [10,17-19].

In this paper we argue that, for the understanding of local malaria ecology on a high resolution scale, an integrated view on multiple influencing factors is helpful, comprising the following objectives:

1. To detect surface water and water-related land cover within the survey region.

2. To assign appropriateness for vector larvae breeding to those land cover types.

3. To investigate the influence of environmental and meteorological variables on larvae and adult vector abundance.

4. To predict the adult *Anopheles *densities in villages using those variables as well as the surrounding densities of larvae.

5. To validate the predicted vectorial risk using ground captured *Anopheles *mosquitoes.

## Methods

**Figure 1:**
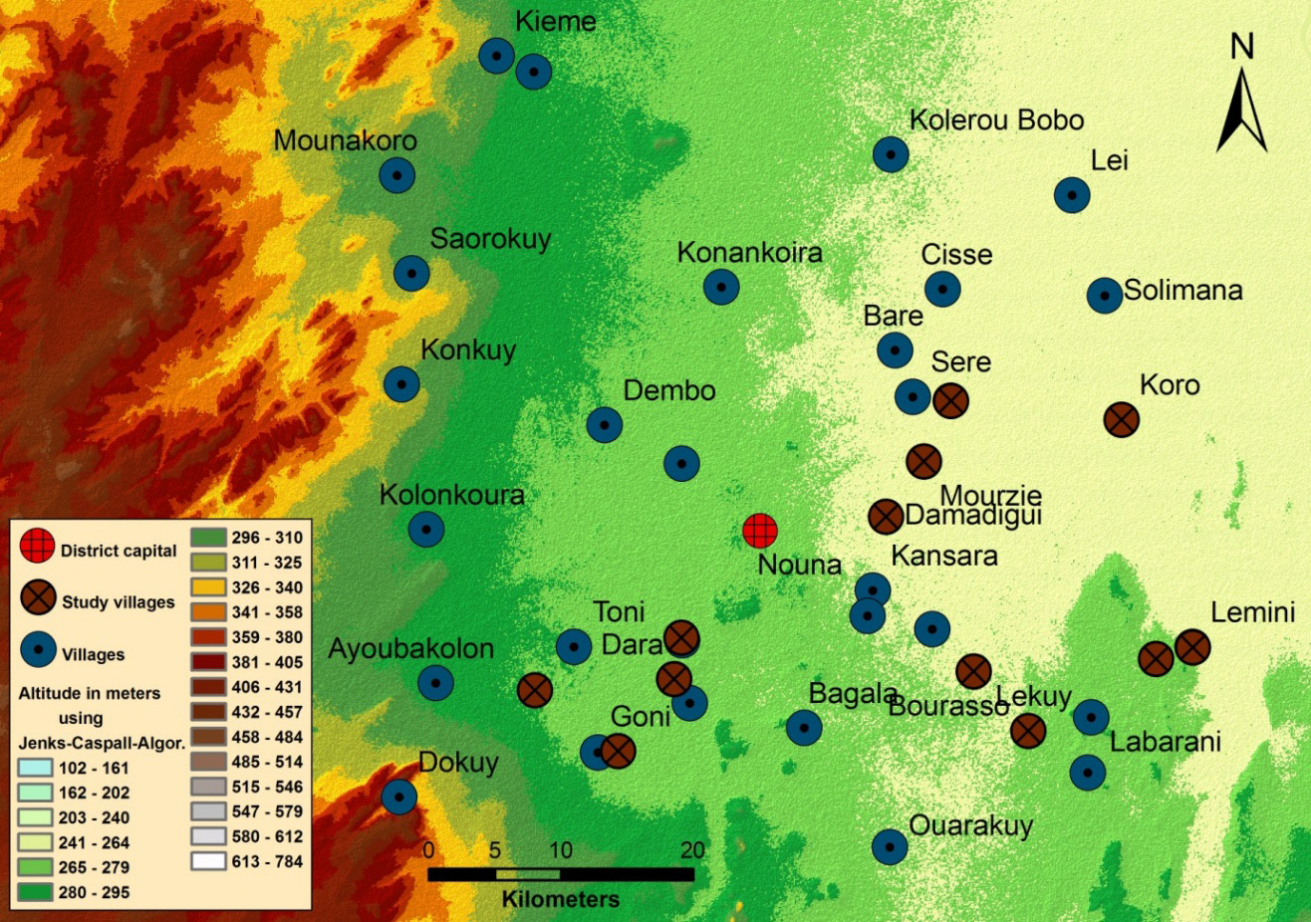
**Digital Elevation Model of the survey region **[20]. Classes were built using natural breaks (Jenks-Caspall-algorithm). The villages and the study villages are presented respectively in blue and dark red.

The study site is located in the North-Western part of Burkina Faso in the Kossi district (Figure 1). The study region shows altitudes around 200 m with increasing elevations towards the West. The mean precipitation for Nouna has been 817 mm per year over a period of the last ten years, with about 90% of annual rainfall during the rainy season (June to September). The monthly maxima during the rainy season can reach up to 350 mm. The yearly average temperature of Nouna is 27.8°C. The North Western savannah regions of Burkina Faso are a malaria holo-endemic area with a marked seasonality. The study took place between July and September 2009, in 12 villages of the region (amongst which 10 were covered by the SPOT-5 satellite scene).

Satellite images from the SPOT-5 (Satellite Pour l'Observation de la Terre) were programmed and acquired for October 12^th ^2009, during the rainy season. Data included three spectral bands at 2.5 m spatial resolution (green, red and near infrared - NIR). One band for the short wave infrared (SWIR) was also available at 10 m spatial resolution. The three first bands were upscaled to 10 m and stacked with the fourth 10 m band. All images were level 3 pre-processed (orthoimages), with map projection UTM zone 30 N, and datum WGS 84. In the center of the satellite scene, which was 60 × 60 km, the district capital Nouna is located at 12° 44' N; 3° 51' W.

A Digital Elevation Model (DEM) at 90 m spatial resolution was available from the Shuttle Radar Topography Mission (SRTM version 4.1) [20,21]. It was resampled at 10 m spatial resolution.

Weekly day and night Land Surface Temperatures (LST) were extracted from MODIS (Moderate Imaging Spectroradiometer) images at 1 km spatial resolution for the full duration of the field work. The freely available MODIS Reprojection Tool [22] was used to extract LST values and to project the resulting images. The weekly LSTs were averaged for the survey region.

The TRMM (Tropical Rainfall Measurement Mission) daily data at 25 km spatial resolution were downloaded for the duration of the survey period including an additional month, for calculating the cumulated precipitations within different time lags before larvae and adult mosquito captures respectively.

**Figure 2:**
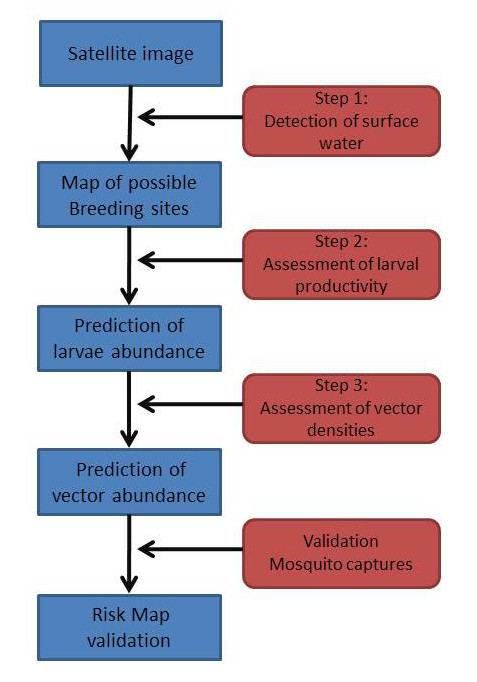
**Technical steps within the approach of Tele-epidemiology**.

The data analysis was based on the conceptual approach of tele-epidemiology developed by CNES, the French Space Agency [23]*i.e*. i- assembling and analyzing multidisciplinary in-situ datasets to identify the main biological and physical mechanisms at stake in order to highlight the main factors implied in the diseases spatial and temporal distribution; ii- remote-sensing monitoring of environment linking the disease with the parameters previously identified with the aim to obtain well adapted products from space; iii- modeling to generate predictive environmental risk maps (Figure 2). This methodology has been previously applied successfully for the Rift Valley Fever in North Senegal [24,25] and for urban malaria in Dakar [26,27]. The basic requirement for malaria to occur is the presence of the vector, *Anopheles*. The abundance of larvae and adults is directly linked to the presence, distribution and persistence of water bodies (puddles, ponds...). Based on this knowledge, the first step of this present study was the detection of surface water (hereafter called step 1). The second part (hereafter called step 2) analyzed the environmental parameters in and around the water collections, that could be related to larvae presence and abundance, and the third step (hereafter called step 3) aimed at identifying the relationships between the predicted larval production, the environmental and meteorological information with the ground recorded *Anopheles *densities, representing a risk indicator for human population.

A Geographic Information System was built in ArcGIS 9.3, containing all remotely sensed and field data. All environmental information was extracted at 10 m pixel level or at village level and transferred into Stata 12 (Stata Corporation, College Station, Texas) for statistical modeling. Multiple indices combining different spectral bands were calculated from the SPOT-5 images using the band math functionality in ENVI 4.7 (Table 1) and tested for statistical association with the presence of surface water, *Anopheles *larvae and imagines abundance according to the three steps above. Land Use and Land Cover (LULC) from a supervised land cover classification were available from a previous study [28].

**Table 1:** Different indices combining different spectral bands were tested for statistical association with presence of surface water in step 1 and for correlation with larvae abundance in step 2

Index	Calculation
NDVI [29] Normalized Difference Vegetation Index	$\frac{NIR-red}{NIR+red}$
SAVI [30] Soil Adjusted Vegetation Index	$\frac{NIR-red}{NIR+red}*\left(1+L\right)$
NDPI [24] Normalized Difference Pond Index	$\frac{SWIR-green}{SWIR+green}$
NDWI Gao [31] Normalized Difference Water Index Gao	$\frac{NIR-SWIR}{NIR+SWIR}$
NDWI Mac Feeters [32] Normalized Difference Water Index Mac Feeters	$\frac{green-NIR}{green+NIR}$
MNDWI Mac Feeters [33] Modified NDWI Mac Feeters	$\frac{green-SWIR}{green+SWIR}$
NDTI [24] Normalized Difference Turbidity Index	$\frac{red-green}{red+green}$

*NIR *near infrared; *SWIR *short wave infrared

### Step 1: Mapping of surface water

Outlines of pools and ponds (locally called "marigots"), in which continuous larvae sampling was performed, were mapped using a GPS device, and transferred to the GIS. Points, further called presence points, were generated in the center of all 10 m pixels that were overlaid by the shape of a water body, even for small areas. Additional points, called absence points, were randomly generated outside of the pools. A total of 482 presence points and 1978 absence points were created. The values of every indicator, as well as the LULC class and the altitude (from the DEM) were extracted at every presence and absence point. The linearity of the relationship between the outcome and each explanatory variable was assessed, except for the elevation that has been included as dichotomous variable. Logistic regressions were fitted to identify the remotely sensed environmental variables significantly associated with the presence/absence of water at each point. The inversion of the best multivariate model allowed generating a 10 m resolution map of the probabilities of presence of water in the studied area.

### Step 2: Mapping of *Anopheles *larvae

**Figure 3:**
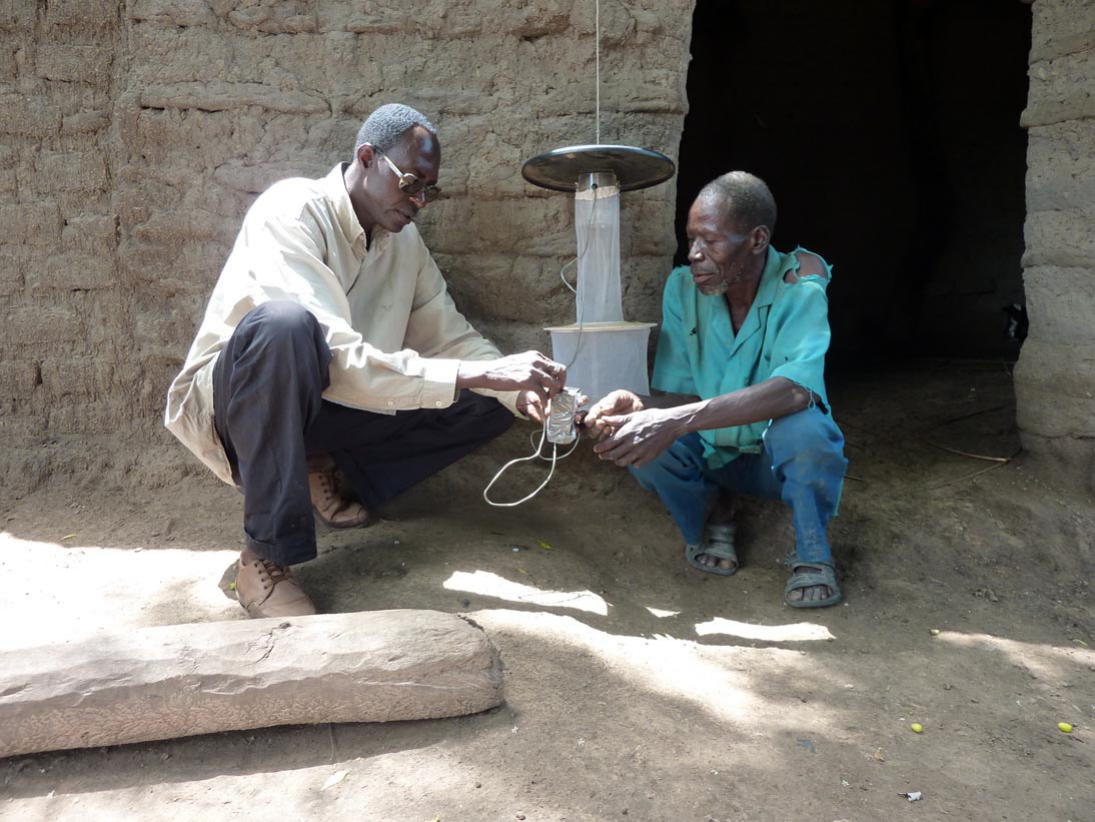
**Installation of a mosquito light trap and giving instructions to an operator in charge for trap surveillance**.

Mosquito larvae at all stages were collected daily from September 2^nd ^to October 23^rd ^2009 in the 12 study villages (2 villages per day with a weekly repetition (Figure 3)). Within and around the villages, the follow-up concerned the most common habitats, *i.e*. the ponds that ranged in size from several meters to hundred meters in diameter. A standardized dipper with 200 ml volume was used to collect larvae.

For each potential breeding site, the environment was taken into account as a mean of the remotely sensed ecological variables computed in and around the water bodies (10 m ring). This scale allowed taking into account the surface (*e.g*. surface cover as vegetation) and nearby structures (*e.g*. shade) on potential habitats that could have an impact on the larval presence and density. Meteorological data was tested at different temporal scales until finding the best statistical association with the larvae presence. The linearity of the relationship between the outcome and each explanatory variable was tested.

A negative binomial regression was fitted to identify those remotely sensed environmental and meteorological variables significantly associated with larval density recorded in each water body at each date. The sampling scheme implied that some correlations could exist between observations obtained in a same water collection since repeated observations could be influenced by similar environmental factors. Yet, the basic requirement for using classical statistics is the independence of observations and neglecting autocorrelations in the analysis may result in overestimation of the strength of the associations. Thus, a pond random effect was added to the models, to account for variables related to the water collection environment that could be significant determinants of the larvae abundance but would not have been measured in the study. The environmental indicators found to be statistically associated with the *Anopheles *larval density were computed and fed back into the GIS, in and around every pond predicted at step 1 of the study in order to predict larvae abundance for the whole study area.

### Step 3: Mapping of adult *Anopheles*

In the 12 villages where the larvae collection took place, adult mosquitoes were captured using light traps. During 9 weeks from September to November 2009 villages were visited within the same time schedule as for the larvae collection, with a repetition period of 6 days. Depending on village accessibility, data was available from 5 to 7 visits. Each day, in 2 villages in the same region light traps were installed. In each village 3 places were chosen where a light trap was placed inside and outside a house respectively. Those pairs of light traps were installed in a distance of approximately 100 m from each other to detect possible local differences in vector abundance between different places within one village. The traps inside the houses were installed near the sleeping places if equipped with an untreated bed net, the traps outside were put beside the house within the common patio, where people stay in the evenings (Figure 3). Light traps were connected to the batteries at 6 pm and disconnected and closed at 6 am. All mosquitoes caught in the light traps were transported to the research centers laboratory for species and gender determination.

The linearity of the relationship between the outcome and each explanatory variable was verified. Then, a negative binomial regression with a village random effect was fitted to predict the density of adult *Anopheles *caught in each village (mean of all the traps) at each date of the ground work using remotely sensed environmental and meteorological predictive variables, as well as larval densities predicted in step 2. As the sampling scheme implied that some correlations could exist between observations from the same village, a random effect was added at the village level.

To predict the adult *Anopheles *densities in non-surveyed villages for every day of the study period, the environmental indicators that were found to be statistically associated with the adult *Anopheles *density were computed in buffers of 500 m around the villages, in accordance with the flight range of emerged mosquitoes from their breeding habitat [34-36].

## Results

### Step 1: Mapping of surface water

A description of the distribution of the remotely-sensed variables used at this step is presented in Table 2. Significant correlations were found in uni- and multivariate analysis between the NDWI Mac Feeters (Normalized Difference Water Index), the NDTI (Normalized Difference Turbidity Index) and the DEM (Digital Elevation Model) amongst 2,460 observations of the presence/absence of ponds at 10 m pixel level (Table 3). The DEM shows differences in altitude between study villages of about 100 m (Figure 1). Even if the contrast of mean elevation between presence and absence points was low, a significant association was highlighted between a low elevation and an increased probability for the presence of ponds. While the NDWI Mac Feeters was positively correlated with the presence of environmental surface water, the NDTI was found to be a protective factor.

**Table 2:** Description of the quantitative remotely-sensed explicative variables associated with the presence of ponds

Variable		Water present	Water absent
	**n observations = 2460**	**n = 482**	**n = 1978**

**NDTI**	Range	-0.25; -0.01	-0.26; 0.05
	Mean and 95% CI	-0.14 [-0.15; -0.15]	-0.10 [-0.10; -0.09]
	25-50-75 percentiles	-0.20; -0.13; -0.10	-0.13; -0.09; -0.06
**NDWI Mac Feeters**	Range	-0.19; 0.42	-0.39; 0.11
	Mean and 95% CI	0.12 [0.11; 0.14]	-0.17 [-0.18; -0.17]
	25-50-75 percentiles	0.03; 0.13; 0.25	-0.21; -0.17; -0.14
**Elevation **In meters	Range	253; 292	253; 292
	Mean and 95% CI	266 [266; 267]	268 [268; 269]
	25-50-75 percentiles	262; 265; 267	261; 268; 274

**Table 3:** Environmental factors significantly associated with the presence of ponds in the 10 meter pixels

	Logistic regression					
	Univariate **			**Multivariate**		

**Number of obs. = 2460**	**Coef**.	95% CI*	**p-value**	**Coef**.	95% CI*	**p-value**

**NDTI**						
Per unit increase	-16.80	-18.91 - -14.69	< 0.0001	-38.81	-43.63 - -28.00	< 0.0001
**NDWI Mac Feeters**						
Per unit increase	34.26	30.55 - 37.98	< 0.0001	43.55	38.54 - 48.77	< 0.0001
**Elevation**						
Inferior to 270 m	1			1		
Superior or equal to 270 m	-1.03	-1.26 - -0.79	< 0.0001	-1.57	-2.22 - -0.92	< 0.0001

* 95% confidence interval

** Only the variables significantly associated in the multivariate model

Logistic regression

Predictions of the probability for the presence of ponds allowed to calculate the area under the ROC (receiver operating characteristic) curve at 0.99 (95% confidence interval: 0.99 - 1.00). The ROC curve is a visualization of the sensitivity, or true positive rate, vs. false positive rate for a binary classifier system whose discrimination threshold is varied. The inversion of the model and the extrapolation for the whole study area allowed generating a map of the probability of presence of water bodies. The application of a cut-off value on those probabilities provided a raster map of the presence/absence of water at 10 m spatial resolution. The filtering (closing filter) and vectorization allowed transforming those maps into maps of ponds. A total of 4,600 water bodies were detected which sizes ranged from 100 to about 5,000 m^2^.

### Step 2: Mapping of *Anopheles *larvae

The *Anopheles *larvae presence/absence (164 observations positive for larvae and 3 negative) and the larval density were recorded during September-November 2009. A total of 16 ponds were digitized in the GIS. All the observations associated with those 16 collections were included in the analysis, for a total of 73 observations. Description of the distribution of the remotely-sensed and meteorological variables used at this step is provided in Table 4. Results of the environmental and meteorological determinants of the *Anopheles *larval density recorded during the field work are presented in Table 5.

**Table 4:** Description of the quantitative remotely-sensed and meteorological explicative variables associated with *Anopheles *larval densities in ponds

Variable		Anopheles larval density* < 28	Anopheles larval density* > = 28 and < 47	Anopheles larval density* > = 47 and < 82	Anopheles larval density* > = 82
	**n observations = 73**	**n = 17**	**n = 19**	**n = 18**	**n = 19**

**NDVI (mean within pond + 10 m ring)**	Range	-0.02; 0.21	-0.12; 0.30	-0.12; 0.30	-0.12; 0.30
	Mean and 95% CI	0.07 [0.04; 0.11]	0.09 [0.04; 0.14]	0.04 [-0.01; 0.10]	0.01 [-0.04; 0.05]
	25-50-75 percentiles	0.00; 0.05; 0.13	0.00; 0.05; 0.21	-0.01; 0.04; 0.05	-0.05; 0.00; 0.05
**Night LST (weekly mean for the survey area) **In °C	Range	20.5; 23.1	20.5; 23.1	20.5; 23.1	21.2; 23.1
	Mean and 95% CI	21.4 [21.0; 21.8]	22.0 [21.6; 22.4]	21.9 [21.5; 22.3]	22.5 [22.2; 22.7]
	25-50-75 percentiles	20.5; 21.1; 21.9	21.1; 22.1; 22.6	21.1; 21.9; 22.7	21.9; 22.6; 23.0

* Larval density = number of larvae per sample (8 dips per pond per date). Categories were chosen following quantiles.

**Table 5:** Meteorological and environmental factors associated significantly with *Anopheles *larval density in ponds

	Negative binomial regression with pond random effect					
	Univariate **			**Multivariate**		

**Number of obs. = 73 Number of ponds = 16**	**Coef**.	95% CI *	**p-value**	**Coef**.	95% CI *	**p-value**
**NDVI** **(mean within pond + 10 m ring)** Per unit increase	-2.64	-4.93 - -0.34	0.024	-3.20	-5.36 - -1.03	0.004
**Night LST** **(weekly mean for the survey area)** Per °C increase	0.33	0.18 - 0.48	< 0.0001	0.36	0.21 - 0.50	< 0.0001

* 95% confidence interval

** Only the variables significantly associated in the multivariate model

Negative binomial regression with pond random effect

Those variables significantly associated with the larval density in multivariate analysis were the mean of the NDVI in and around the ponds and the current night Land Surface Temperature (night LST). The NDVI was negatively associated whereas the LST was positively associated with the *Anopheles *larval density in ponds.

The results of the likelihood ratio test (*p *< 0.001) showed that the random effect model was significantly different from a model fitted without accounting for the pond effect. Predictions of the larval densities for each observation allowed the calculation of Spearman correlation with the observed densities at 0.55, showing medium correlation between the tested variables. Daily maps were drawn by inverting the global model predicting the *Anopheles *larval density for each water collection detected at step 1, for the full duration of the follow-up. Those maps were used as basis of step 3.

### Step 3: Mapping of adult *Anopheles*

**Figure 4:**
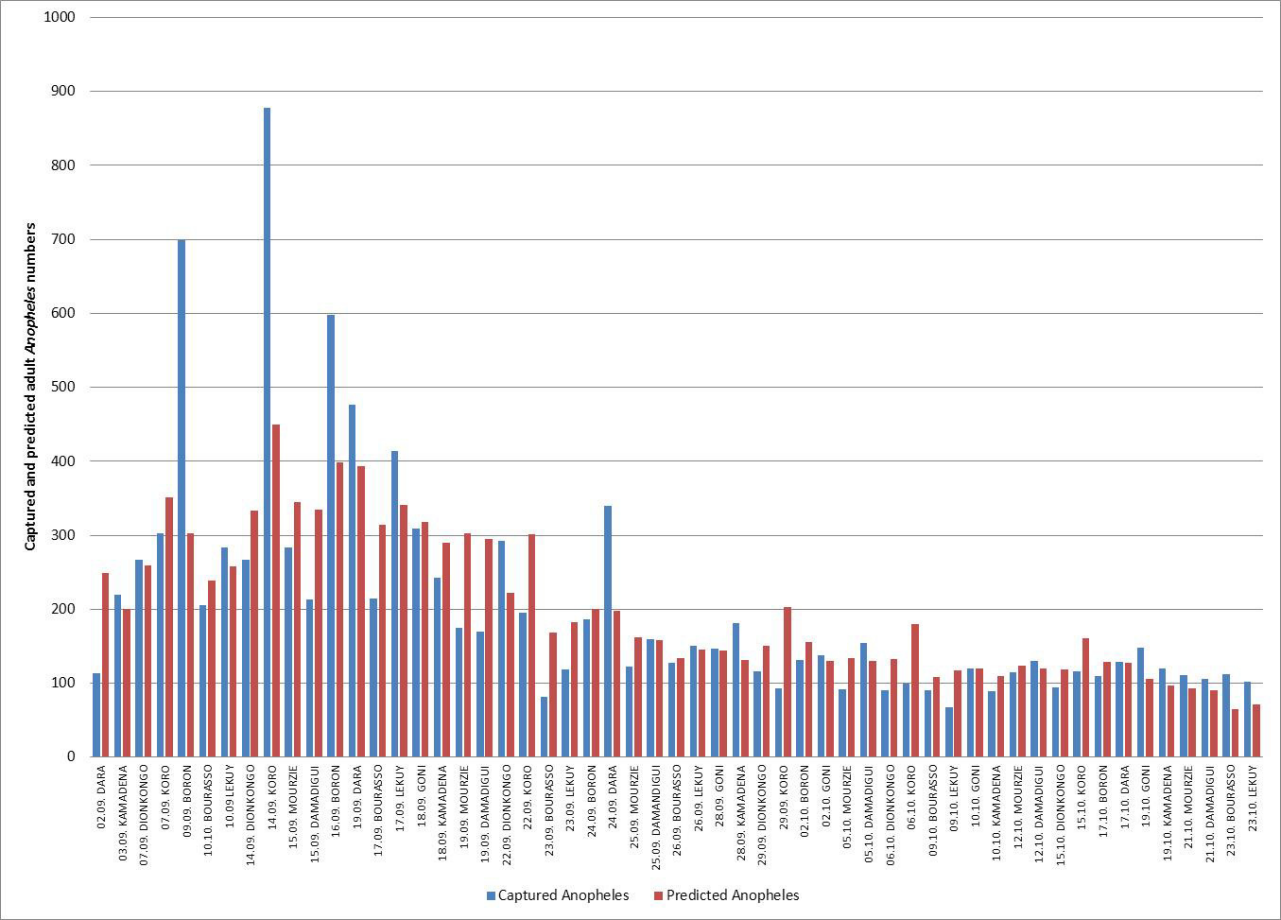
**Captured (blue) and predicted (red) Anopheles numbers for 10 study villages with continuous larvae sampling and position of buffer zone within the satellite scene for the duration of mosquito captures (2nd September - 23 rd October 2009)**.

**Figure 5:**
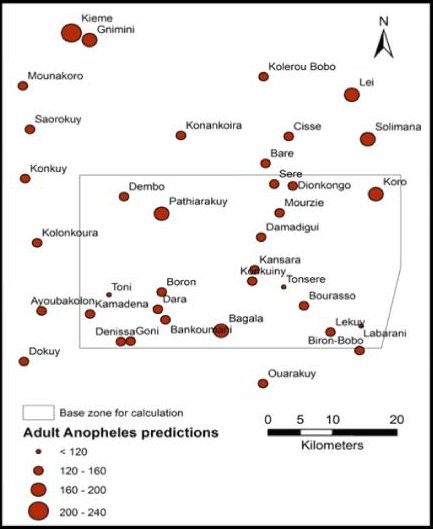
**Adult Anopheles predictions for 37 villages within the satellite scene of SPOT 5 for the 1st October 2009**. Data used for the predictions in all 40 villages have been derived from villages within the "base zone for calculations", the zone in which data was taken during fieldwork.

During the study period, 99% of the *Anopheles *caught in the traps were *Anopheles gambiae s.l. and *1% *Anopheles funestus*. For both species, 89% of caught mosquitoes were female, 11% were male. The total larval production (surface water predicted in step 1 multiplied by the larval density predicted in step 2 for a given date) in the buffers around the villages was not significantly associated with the number of *Anopheles *caught in the traps. None of the LULC classes extracted from the supervised classification was associated with the adult densities. Nevertheless, other environmental (NDPI) and meteorological (day LST and rainfall amount) factors were significantly associated with the adult densities (Tables 6 and 7). On the basis of the multivariate negative binomial regression, adult *Anopheles *predictions for the 10 study villages covered by the SPOT image were made for all capture dates between September 2^nd ^and October 23^rd ^2009. The comparison with actual values is presented in Figure 4. Figure 5 is the prediction map of *Anopheles *vector densities for October, 1^st^, 2009, for the 37 villages included in the SPOT-image outline.

**Table 6:** Description of the quantitative remotely-sensed and meteorological explicative variables associated with the adult *Anopheles *densities in villages

Variable		Anopheles adult density* < 110	*Anopheles adult density > = 110 and < 145*	*Anopheles adult density > = 145 and < 240*	*Anopheles adult density > = 240*
	**n observations = 55**	**n = 12**	**n = 15**	**n = 14**	**n = 14**

**NDPI** **(mean in 500 m buffer around study villages)**	Range	0.06; 0.13	0.06; 0.13	0.06; 0.13	0.06; 0.13
	Mean and 95% CI	0.09 [0.07; 0.10]	0.08 [0.08; 0.09]	0.08 [0.07; 0.09]	0.09 [0.08; 0.10]
	25-50-75 percentiles	0.07; 0.08; 0.09	0.07; 0.09; 0.10	0.07; 0.08; 0.08	0.08; 0.09; 0.10
**Day LST(weekly mean for the survey area)** In °C	Range	27.0; 33.0	26.2; 33.0	23.1; 30.6	23.1; 27.7
	Mean and 95% CI	29.9 [29.0; 30.9]	29.6 [28.8; 30.4]	26.9 [25.6; 28.2]	24.5 [23.8; 25.3]
	25-50-75 percentiles	29.5; 29.8; 30.4	28.7; 30.0; 30.3	24.4; 27.7; 28.7	23.5; 23.8; 25.4
					
**Rainfall amount (sum during 15 preceding days for each village)** In mm	Range	22.1; 92.0	15.3; 147.4	22.1; 168.4	38.1; 220.2
	Mean and 95% CI	55.2 [39.6; 70.8]	51.9 [34.0; 69.7]	91.2 [64.3; 118.2]	145.7 [114.8; 176.6]
	25-50-75 percentiles	36.6; 46.5; 78.7	25.6; 38.1; 73.6	41.2; 83.3; 131.2	131.3; 148.8; 175.3

**Table 7:** Meteorological and environmental factors associated with adult *Anopheles *abundance in Nouna region in September-November 2009

	Negative binomial regression with village random effect					
	**Univariate**			**Multivariate**		

**Number of obs. = 55** **(Nights withmosquito captures) Number of villages = 10**	**Coef**.	95% CI *	**p-value**	**Coef**.	95% CI *	**p-value**

**NDPI** **(mean in 500 m buffer around study villages)**						
Per unit increase	6.01	-1.36 - 13.38	0.110	7.52	0.77 - 14.27	0.029
**Day LST** **(weekly mean for the survey area)**						
Per °C increase	-0.16	-0.19 - -0.12	< 0.0001	-0.16	-0.20 - -0.12	< 0.0001
**Rainfall amount (sum during 15 preceding days for each village)**						
Per 10 mm increase	0.06	0.04 - 0.08	< 0.0001			NS

* 95% confidence interval

Binomial negative regression with village random effect. Univariate and multivariate analysis

## Discussion

The present study allowed drawing predictive high-resolution risk maps for malaria vector abundance in a rural area following three modeling steps: the detection of water bodies, the larvae abundance in those water bodies and the adult *Anopheles *densities in villages. In the multivariate models for all three study steps, there was more than one relevant influencing factor for the presence of surface water as well as for *Anopheles *larvae and imagines. Due to the close interweavement of factors influencing the surface water presence and vector larvae and imagines abundance, the number of used and combined techniques is comparably higher than in studies that focus on a single step, *e.g*. detection of appropriate larval habitats or prediction of vector density.

### Step 1: Mapping of surface water

The digital elevation model turned out to be an indicator for surface water presence and swampy areas even in the survey region's setting with little differences in local altitudes, while from survey regions that are more heterogeneous and hilly this correlation and even its influence on malaria parasite prevalence has already been stated [37]. The NDWI Mac Feeters was positively correlated with the presence of water, in line with the fact that it is an indicator that increases with open water presence, having been previously used for water detection [26,27]. The NDTI, initially designed to describe water turbidity, increases when water bodies become muddy and have spectral reflectance similar to bare soils [24] so it was logically negatively associated with the presence of water.

The smallest pond recorded on the ground covered one 10 m pixel so the direct water detection - using indices thresholds coupled with photointerpretation [24,25] - was not appropriate. Indeed, it is commonly stated that object detection is feasible only when the object size is at least 1.5 times larger than the pixel size. Instead, statistical modelling allowed to benefit from several remotely sensed data sources that could be put together to predict the probability of presence of ponds. This methodology already proved to be efficient for detection of small *Anopheles *breeding sites in urban settings [27]. The pixels that were predicted as belonging to water bodies were grouped into water collections as single objects, as it has been done in the North of Senegal for the mapping of ponds harbouring larvae for Rift Valley Fever vectors [24,25]. Indeed, important predictors that could further be related to the presence of larvae, such as shade around the collection, were water body-related. In consequence, they had to be mapped at the level of the water collection and not at the pixel-level.

Step1 took advantage of the availability of a high resolution SPOT image that was acquired at the time of the field collections and that allowed to predict the presence of relatively small ponds recorded on the ground. As the image was taken during the rainy season, maximum water collections were predicted, that could further be weighted depending on the season. Even at 90 m spatial resolution, the DEM provided useful information for mapping the surface water and it should be expected that a DEM with an increased spatial resolution would improve the prediction by highlighting small altitude contrasts.

### Step 2: Mapping *Anopheles *larvae

The findings were generally in line with the findings of other studies but show some particularities due to the very high resolution remote sensing approach. The NDVI at this scale turned out to be a hindering factor for larval production in contrast to most studies that utilize satellite imagery at a lower scale [38-40]. As far as the authors are aware, this correlation is unique to this study but may be reverse if changing towards a different scale. In the present study, the NDVI could have been a proxy of shade on the water collection or surface vegetation that are both usually related to lower *Anopheles *larval densities [41-43]. In consequence, the negative association between NDVI and the larval density was coherent with the biological mechanisms of larval development already highlighted in ground studies.

The night LST during the ground prospecting days was significantly positively related to the *Anopheles *larval densities in the water collections. In breeding sites, the entire development cycle from egg to the emerging imago can be completed under favorable conditions. This cycle's duration can vary between 1 and 3 weeks, depending on water and air temperatures, assuming sufficient food availability. Its length shortens with temperature increase [44] in accordance with the observation that LST was significantly positively associated with the density of larvae. Night LST showed stronger correlation than day temperature, probably due to the decreased impact of solar radiation and a more representative portrait of the average water temperature.

The cumulated rainfall during the 15 days preceding the day of larvae collection was positively associated with the low/high densities of larvae (results not shown) in univariate analysis. Rainfall patterns steer the availability of surface water for mosquito larvae breeding. In this study, only flooded water collections were included, so rainfall was already taken into account by definition. Nevertheless, the amount of rainfall could be a proxy for the persistence of water bodies, providing sufficient time for larvae to develop. On the other hand, rainfall might be a surrogate variable for global larval productivity within a region.

Step 2 benefited from the high spatial resolution of the SPOT image and the high temporal resolution of the meteorological data. Indeed, rainfall and temperature were taken into account at coarse spatial resolution (village and study area levels) which was consistent with scales of the meteorological heterogeneity. Nevertheless, the daily or weekly repetition of data was necessary for evaluating the evolutions of local conditions favourable for larvae development.

### Step 3: Mapping adult *Anopheles*

The multivariate model predicting the *Anopheles *adult densities at the village level included an environmental and a meteorological variable, which allowed recording of a spatial (using NDPI in 500 m buffers around the villages) effect that differed between villages and a temporal effect (using weekly LST).

The NDPI was correlated with the number of captured mosquitoes. Increasing values for the NDPI characterize the presence of mixed pixels that show both, water and vegetation. The presence of this mixed environment can be seen as an ideal prerequisite for emerging imagines to rest. In consequence, the NDPI was logically associated positively with the *Anopheles *adult densities.

As a meteorological factor, night LST was not significantly associated with the adult mosquito density whereas day LST was negatively associated. The higher the day LST, the lower the number of mosquitoes caught, which may indicate higher environmental stress, making it more difficult to survive or search for blood meal. Higher day LSTs in this region also go hand-in-hand with a lower relative humidity, especially during the rainy season. Lower environmental air humidity is less appropriate for mosquitoes survival [45] and blood meal retrieval success [46].

The cumulative amount of rainfall in the 15 preceding days was significantly positively correlated with the adult *Anopheles *density in univariate analysis. This is likely to be explained by the increased number and extended outlast of larval habitats. It can also be an indicator of humid air conditions that favors adult mosquito survival.

The prediction for larval densities in environmental habitats within the buffer zone around mosquito capture points (500 m) was not significantly correlated with the imagines abundance. In extrapolating the prediction models for water collections and larval densities to assess adult *Anopheles *densities, some errors were accepted, such as follows.

• Randomly created points for the absence of surface water did possibly fall to some extent into zones that were covered with water or had high soil humidity. In consequence, the map of water collection could have been biased. Here, a more detailed study of the situation around the study villages would be needed, with the collection of ground absence points (*i.e*. absence of water).

• The quality of the validation of the prediction of larval densities with actual ground values was moderate, larger field dataset collections may improve the accuracy of the models.

• Working with a single satellite image, the positions and in particular the dimensions of ponds and other surface water were static and did not take into consideration the possible change in circumference after rainfalls or longer periods of continuous evaporation. The acquisition of several images could improve this point, as well as the modeling of the size of the ponds depending on rainfall amount and distribution as it has been done in North Senegal [25].

• Step 2 was undertaken to model larval densities, as no multivariate model could have been adjusted to predict the presence/absence of larvae. In consequence, the water collections that were not breeding sites could have been misclassified.

### Ground entomological data

The field work undertaken in the Nouna region in the 2009 rainy season showed that most of the *Anopheles *caught in traps were *An. gambiae s.l*. that are known to be vectors for malaria. In consequence, risk maps drawn in the present study, may be seen as basis information for malaria risk mapping as the location of the vector's larval habitats and their dynamics are the primary determinants of the spatial and temporal distribution of malaria transmission. Then, in addition to this entomological approach, it should be emphasized that malaria transmission occurs only if a competent infected vector meets a sensitive human population; if a *Plasmodium *reservoir is present.

Data was collected from September to November, the period representing the peak and outgoing rainy season. Onset of precipitations is volatile during the last years but usually starts in June. The adult vector abundance, and with some delay malaria transmission find their peak around September. In consequence, the risk predicted in the present study can be seen as the maximum annual entomological malaria risk. Even given the much lower malaria transmission during more than 6 months due to drastically reduction of environmental habitats and rainfall, an extended future study including more survey month could allow to better understand the year round vector dynamics. Still little is known about the vector resting places during dry season and the process of re-emergence in rainy season.

Only the ponds have been followed-up in and around the study villages while small water collections that are known to be seasonally productive breeding sites of *An. gambiae s.l*. were not included for logistical reasons. It is argued that the larval productivity recorded at pond level may have been a surrogate evaluation of the total productivity in the villages. Nevertheless, it cannot be excluded that the lack of association between larval and adult densities may partly rely on this partial sampling. In addition, the use of light traps to catch adult mosquitoes may have biased the estimates of vector densities - *e.g*. depending on the density [47] and have introduced noise that could also explain the absence of significant association between the larval and adult densities.

In general, differences in vector abundance between villages may not only be subject to environmental, but also anthropogenic variables. Within the study area, there were no vector control interventions performed during the study year and the use of bed nets was equally distributed. In consequence, no bias was introduced when evaluating entomological figures.

## Conclusion

Remotely sensed environmental and meteorological data allowed the prediction of water presence in the region of Nouna, as well as the dynamic prediction of *Anopheles *larval and adult densities. While high resolution satellite data provided possibilities for spatial mapping of vector abundance, the amount and regional distribution of precipitations and the temperature are the drivers for vector development; the temporal component of the risk model. The results of the present study could be seen as the basic element of a dynamic system aiming at facilitating real-time monitoring of human health in rural Burkina Faso. The derived risk maps may keep validity for several years up to a decade, since the geographic factors change only within small limits. Having ground truth data on the region's characteristic environmental features, the extension of this approach to neighboring regions should be possible with a significantly reduced need for preparatory fieldwork. With the acquisition of additional satellite images and weather data, predictions on vector abundance for bordering areas can be performed. For usage in additional regions, the techniques appliance can be probably performed with drastically decreased costs due to the omission of extensive fieldwork components.

## Competing interests

The authors have not received any funding or benefits from industry to conduct this study.

## Authors' contributions

PD contributed to the conception and the design of the study, collected the field data and contributed to the analysis and interpretation of the data and to the writing of the paper. VM contributed to the study design, the analysis and interpretation of the data and wrote the paper. JPL contributed to the conception and the design of the study and the interpretation of the data. CV contributed to the analysis and interpretation of the data. AS contributed to the conception and the design of the study. RS contributed to the conception and the design of the study. All authors read and approved the final manuscript.
